# Responses of primary osteoblasts and osteoclasts from hemizygous β-globin knockout thalassemic mice with elevated plasma glucose to 1,25-dihydroxyvitamin D_3_

**DOI:** 10.1038/s41598-019-50414-7

**Published:** 2019-09-27

**Authors:** Narattaphol Charoenphandhu, Ratchaneevan Aeimlapa, Supagarn Sooksawanwit, Jirawan Thongbunchoo, Jarinthorn Teerapornpuntakit, Saovaros Svasti, Kannikar Wongdee

**Affiliations:** 1 Department of Physiology, Faculty of Science, Mahidol University, Bangkok, 10400 Thailand; 2 Center of Calcium and Bone Research (COCAB), Faculty of Science, Mahidol University, Bangkok, 10400 Thailand; 3 Institute of Molecular Biosciences, Mahidol University, Nakhon Pathom, 73170 Thailand; 4 The Academy of Science, The Royal Society of Thailand, Dusit, Bangkok, 10300 Thailand; 5 Department of Physiology, Faculty of Medical Science, Naresuan University, Phitsanulok, 65000 Thailand; 6 Thalassemia Research Center, Institute of Molecular Biosciences, Mahidol University, Nakhon Pathom, 73170 Thailand; 7 Faculty of Allied Health Sciences, Burapha University, Chonburi, 20131 Thailand

**Keywords:** Ion transport, Calcium and vitamin D

## Abstract

β-thalassemia is often associated with hyperglycemia, osteoporosis and increased fracture risk. However, the underlying mechanisms of the thalassemia-associated bone loss remain unclear. It might result from abnormal activities of osteoblasts and osteoclasts, and perhaps prolonged exposure to high extracellular glucose. Herein, we determined the rate of duodenal calcium transport in hemizygous β-globin knockout thalassemic (BKO) mice. Their bones were collected for primary osteoblast and osteoclast culture. We found that BKO mice had lower calcium absorption than their wild-type (WT) littermates. Osteoblasts from BKO mice showed aberrant expression of osteoblast-specific genes, e.g., Runx2, alkaline phosphatase and osteocalcin, which could be partially restored by 1,25(OH)_2_D_3_ treatment. However, the mRNA expression levels of RANK, calcitonin receptor (Calcr), c-Fos, NFATc1, cathepsin K and DMT1 were similar in both BKO and WT groups. Exposure to high extracellular glucose modestly but significantly affected the expression of osteoclast-specific markers in WT osteoclasts with no significant effect on osteoblast-specific genes in WT osteoblasts. Thus, high glucose alone was unable to convert WT bone cells to BKO-like bone cells. In conclusion, the impaired calcium absorption and mutation-related aberrant bone cell function rather than exposure to high blood glucose were likely to be the principal causes of thalassemic bone loss.

## Introduction

β-thalassemia—a hereditary anemic disease caused by loss-of-function mutation of β-globin gene^1^—has been reported to cause a number of complications, including ineffective erythropoiesis, growth retardation, osteoporosis, hyperglycemia and diabetes mellitus (DM)^2^. Our previous investigations in hemizygous β-globin knockout thalassemic (BKO) mice indicated that the thalassemia-associated bone loss was partially caused by chronic exposure to inflammatory cytokines [e.g., interleukin (IL)−1α and −1β)], which tremendously enhanced osteoclastogenesis and osteoclast-mediated bone resorption and decreased bone formation^3^. Thalassemic mice also exhibited higher rates of intestinal iron transport and expression of iron transporters [e.g., divalent metal transporter (DMT) − 1]^4,5^. It was interesting to note that iron overload, dysregulation of iron metabolism and abnormal expression of iron transporters [e.g., DMT1 and ferroportin-1] also enhance inflammation and release of cytokines from several cell types^6,7^. Nevertheless, despite the signs of impaired osteoblast functions^8,9^, how thalassemia altered the osteoblast-specific gene expression and phenotype remains elusive.

Generally, osteoblasts that were exposed to hyperglycemic condition would have impaired functions, such as decreased expressions of alkaline phosphatase (ALP), osteocalcin, and osteopontin^10,11^. Since thalassemic patients often manifest high plasma glucose that may develop DM in adulthood, and since DM is known to associate with osteoporosis and poor bone strength^10,12–14^, it is possible that exposure to high extracellular glucose might also aggravate the impairment of bone cell function under thalassemic condition. Alternatively, it was also hypothesized that osteoblasts and/or osteoclasts from thalassemic mice probably malfunctioned from their genetic background. In other words, the thalassemic bone cells may exhibited abnormalities by themselves in the absence of proinflammatory cytokine exposure or high glucose.

In addition, thalassemic patients were reported to have lower circulating levels of calciotropic hormones, 25-hydroxyvitamin D [25(OH)D] and 1,25-dihydroxyvitamin D_3_ [1,25(OH)_2_D_3_]^15,16^, the latter of which is a potent stimulator of intestinal calcium absorption and modulator of bone cell differentiation^17,18^. Thus, thalassemic mice were expected to show an impairment of calcium absorption that perhaps indirectly contributed to aberrant bone cell functions. In that case, 1,25(OH)_2_D_3_ treatment should alleviate the thalassemia-associated impairment of osteoblast and osteoclast functions.

Therefore, the objectives of the present study were (*i*) to confirm the presence of aberrant calcium metabolism in BKO versus wild-type (WT) mice; (*ii*) to determine the mRNA expression of osteoblast- and osteoclast-related markers in primary osteoblasts and osteoclasts derived from BKO mice; (*iii*) to find out whether 1,25(OH)_2_D_3_ was able to rescue the expression of bone markers; and (*iv*) to investigate the responses of primary osteoblasts and osteoclasts from WT mice to high extracellular glucose and observe whether they exhibited similar abnormalities as BKO bone cells or were converted to BKO-like cells. The BKO mice used in the present study normally exhibited thalassemia intermedia featuring congenital anemia with microcytosis, hypochromasia, anisopoikilocytosis, ineffective erythropoiesis, consistent with the phenotype observed in β-thalassemic patients^19^. We used 9-week-old female mice for isolating primary bone cells because our previous *in vivo* study has demonstrated that they clearly manifested osteopenia after 8 weeks of age as assessed by dual-energy x-ray absorptiometry and bone histomorphometry^3^.

## Results

### BKO mice exhibited the impaired intestinal calcium absorption and glucose metabolism

**Figure 1:**
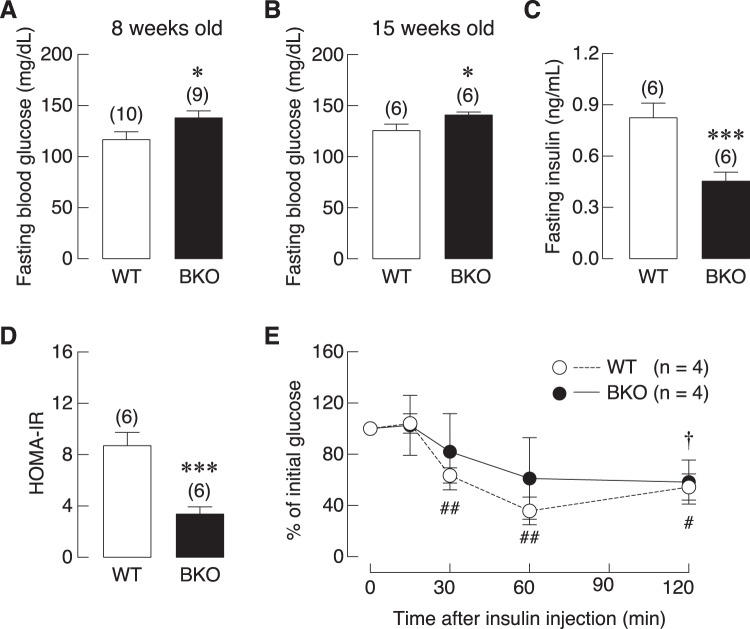
Blood glucose and insulin profiles derived from 8- and 15-week-old BKO and WT mice, i.e., (**A**,** B**) Fasting blood glucose from 8- and 15-week-old mice, (**C**) fasting insulin level of 15-week-old mice, (**D**) homeostatic model assessment of insulin resistance (HOMA-IR) index of 15-week-old mice, *P < 0.05, ***P < 0.001 vs. age-matched WT mice, (**E**) percentage of initial glucose derived from insulin tolerance test (ITT) of 8-week-old mice. ^#^*P* < 0.05, ^##^*P* < 0.01 vs. BKO mice, ^†^*P* < 0.05. vs. 0 min after insulin injection of BKO mice. Numbers in parentheses are numbers of animals.

Consistent with our previous report of lower serum levels of 1,25(OH)_2_D_3_ in BKO mice^5^, we were able to demonstrate the impairment of intestinal calcium transport in both male and female BKO mice, as shown in Table 1. However, the duodenal electrical parameters, i.e., transepithelial potential difference (PD), short circuit current (*I*sc) and transepithelial resistance (TER) of BKO mice were comparable to those of WT mice (Table 1). Bone histomorphometric analysis also confirmed that male BKO mice manifested several signs of suppressed osteoblast functions using proxy indicators of osteoblast activity, extracellular matrix accumulation and mineralization, i.e., decreases in osteoblast surface, osteoid surface, osteoid volume, mineral apposition rate, double labeled surface and bone formation rate, as well as the enhanced osteoclast activity, i.e., increases in osteoclast surface and active erosion surface (Supplementary Fig. S1). In addition, similar to some thalassemic patients with iron overload-induced insulin resistance^12^, both 8- and 15-week-old BKO mice exhibited higher fasting blood glucose, a sign of abnormal glucose metabolism. They also had an impaired insulin tolerance and lower fasting insulin level despite a lower HOMA index (Fig. 1).

**Table 1:** Transepithelial calcium flux and electrical parameters of the duodenal epithelia in male and female WT and BKO mice.

Experimental Groups	Transepithelial calcium flux, nmol h^−1^ cm^−2^	Electrical parameters		
		PD, mV	*I*sc, μA·cm^−2^	TER, Ω·cm^2^
**Male**				
WT	13.44 ± 1.69	1.08 ± 0.29	13.67 ± 7.06	120.00 ± 17.34
BKO	9.08 ± 1.01*	3.23 ± 1.20	28.17 ± 10.24	130.20 ± 23.34
**Female**				
WT	17.55 ± 1.30	1.79 ± 0.40	16.78 ± 2.44	109.80 ± 11.32
BKO	7.06 ± 0.65***	1.58 ± 0.35	11.67 ± 1.81	142.80 ± 13.73*

Values are means ± SE. Numbers of animals per group are five to nine animals (n = 5–9). PD, transepithelial potential difference; *I*sc, short circuit current; TER, transepithelial resistance. *P < 0.05 and ***P < 0.001 compared with sex- and age-matched WT group.

### Primary osteoblasts from BKO mice showed aberrant expression of osteoblast-specific genes, which could be partially restored by 1,25(OH)_2_D_3_

**Figure 2:**
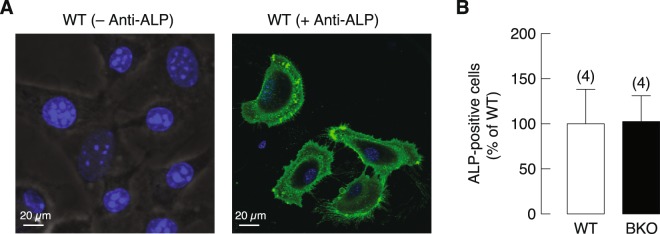
(**A**) A representative immunofluorescence photomicrograph shows expression and localization of ALP protein in primary osteoblasts isolated from wild-type (WT) mice (60 × magnification). The primary osteoblasts were plated on coverslip and incubated with primary antibody against ALP (+Anti-ALP), the negative control was incubated with normal goat IgG (−Anti-ALP). The cells were stained for ALP (green) and nuclei (blue). (**B**) Quantitative ALP-positive cells in primary osteoblasts which represented as percentage of WT control. Numbers in parentheses are numbers of animals.

ALP is widely used as an early marker of osteoblast differentiation both *in vivo* and *in vitro*^20^. In the present study, ALP antibody was used to identify the UMR-106 osteoblast-like cell line by using flow cytometry (Supplementary Fig. S2) and ALP expression was detected in primary osteoblasts of WT mice (Fig. 2A). Flow cytometric analysis revealed that there was no difference in the number of ALP-positive cells in primary osteoblast cultures from WT and BKO mice (Fig. 2B).

**Figure 3:**
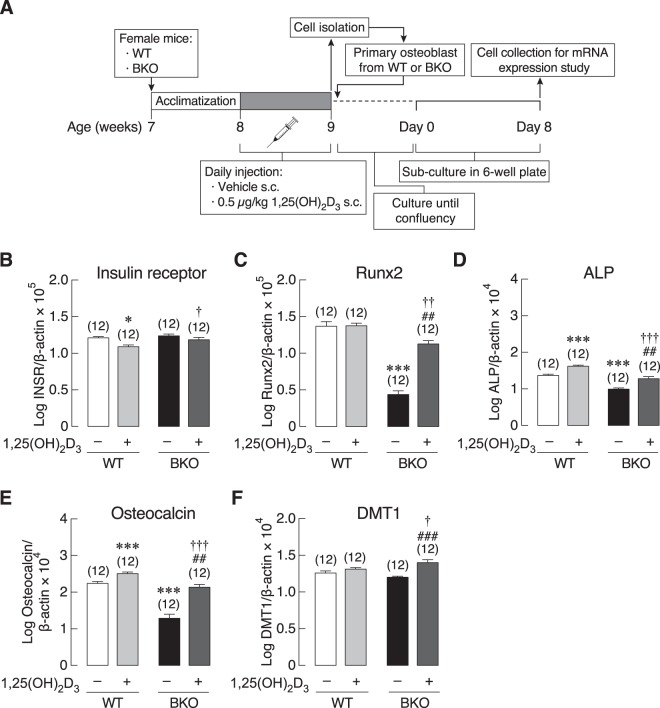
(**A**) A schematic picture shows primary osteoblast collection plan for mRNA expression study. Primary osteoblasts were isolated from 0.5 μg/kg 1,25(OH)_2_D_3_-treated or 3 mL/kg 9:1 propylene glycol-ethanol (vehicle)-treated BKO and WT mice. The mRNA expression levels of osteoblast-specific markers, i.e., (**B**) insulin receptor (INSR), (**C**) Runx2, (**D**) alkaline phosphatase (ALP), (**E**) osteocalcin, and (**F**) divalent metal transporter (DMT) 1 in 8-day primary osteoblasts culture of BKO and WT mice. β-actin was a housekeeping gene for normalization. *P < 0.05, ***P < 0.001 vs. vehicle-treated WT mice (white bar), ^##^*P* < 0.01, ^###^*P* < 0.001 vs. vehicle-treated BKO mice (black bar), ^†^*P* < 0.05, ^††^*P* < 0.01, ^†††^*P* < 0.001 vs. 1,25(OH)_2_D_3_-treated WT mice. Numbers in parentheses represent the number of independent samples per group.

**Figure 4:**
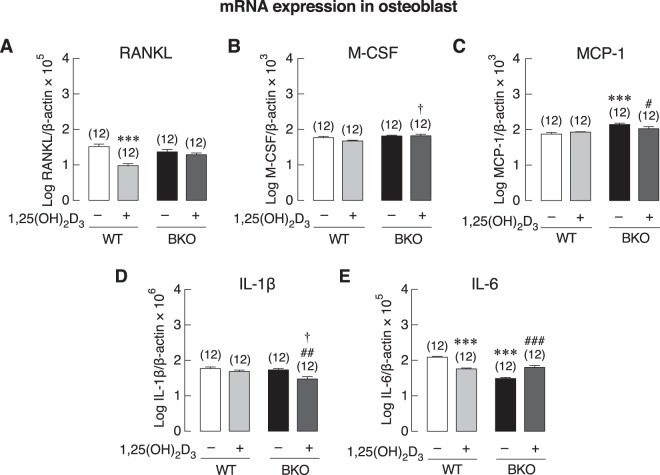
mRNA expression levels of osteoblast-derived osteoclastogenesis-related genes, i.e., (**A**) RANKL, (**B**) M-CSF, (**C**) MCP-1, (**D**) IL-1β, and (**E**) IL-6 in 8-day primary osteoblast culture from BKO and WT mice. Primary osteoblasts were isolated from 0.5 μg/kg 1,25(OH)_2_D_3_-treated or 3 mL/kg 9:1 propylene glycol-ethanol (vehicle)-treated BKO and WT mice. β-actin was a housekeeping gene for normalization. ***P < 0.001 vs. vehicle-treated WT mice (white bar), ^#^*P* < 0.05, ^##^*P* < 0.01, ^###^*P* < 0.001 vs. vehicle-treated BKO mice (black bar), ^†^*P* < 0.05 vs. 1,25(OH)_2_D_3_-treated WT mice. Numbers in parentheses represent the number of independent samples per group.

Nevertheless, despite similar cell number in the two primary osteoblast cultures, the mRNA expression levels of several osteoblast-specific markers, i.e., Runx2 (transcription factor determining osteoblast differentiation), ALP (differentiation marker) and osteocalcin, were significantly lower in primary osteoblasts of BKO than WT mice (Fig. 3). The function or importance of each marker was briefly mentioned in Supplementary Table S1. Furthermore, since DMT1 is one of the major transporters for cellular iron uptake, which can induce iron toxicity and trigger cellular response during iron overload^21^, the expression of DMT1 was determined in the present study. As shown in Fig. 3F, BKO osteoblasts showed the same level of DMT1 expression compared with WT. However, mRNA expression of osteoblast-derived osteoclastogenesis-related genes in primary osteoblasts from BKO mice showed upregulation of MCP-1 and downregulation of IL-6 (Fig. 4) while RANKL, M-CSF, and IL-1β expressions remained unchanged (Fig. 4).

In some experiments, the osteoblasts were collected from mice pretreated subcutaneously for 7 days with 0.5 μg/kg of 1,25(OH)_2_D_3_, as shown in the timeline (Fig. 3A). We found that 1,25(OH)_2_D_3_ pretreatment reduced the expression of insulin receptor (INSR) in primary osteoblasts from WT, but increased INSR expression in cells from BKO mice when compared with 1,25(OH)_2_D_3_-treated WT group (Fig. 3B). Interestingly, 1,25(OH)_2_D_3_ was able to restore the mRNA expression of Runx2, ALP and osteocalcin, and upregulated the DMT1 expression in BKO mice (Fig. 3). As expected 1,25(OH)_2_D_3_ downregulated the expression of RANKL and IL-6 in cells from WT group, but modestly upregulated the expression of IL-6 while downregulating IL-1β and MCP-1 expression in cells from BKO mice (Fig. 4).

### BKO genotype decreased the number of TRAP-positive cells in primary osteoclast culture

**Figure 5:**
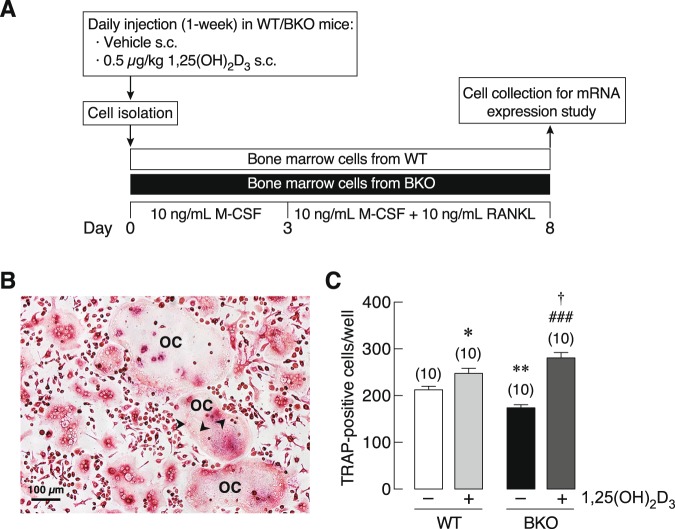
(**A**) A schematic picture shows primary osteoclast collection plan for mRNA expression study. Primary osteoclasts were isolated from 0.5 μg/kg 1,25(OH)_2_D_3_-treated or 3 mL/kg 9:1 propylene glycol-ethanol (vehicle)-treated BKO and WT mice. During 8-day culture, 10 ng/mL M-CSF and 10 ng/mL RANKL were added in culture media to induce osteoclast differentiation. (**B**) Representative photomicrographs of TRAP-positive multinucleated osteoclasts (OC). Arrowhead, nucleus of osteoclast. Scale bar, 100 µm. (**C**) Numbers of TRAP-positive cells/well. *P < 0.05, **P < 0.001 vs. vehicle-treated WT mice (white bar), ^###^*P* < 0.001 vs. vehicle-treated BKO mice (black bar), ^†^*P* < 0.05 vs. 1,25(OH)_2_D_3_-treated WT mice. Numbers in parentheses represent the number of independent samples per group.

**Figure 6:**
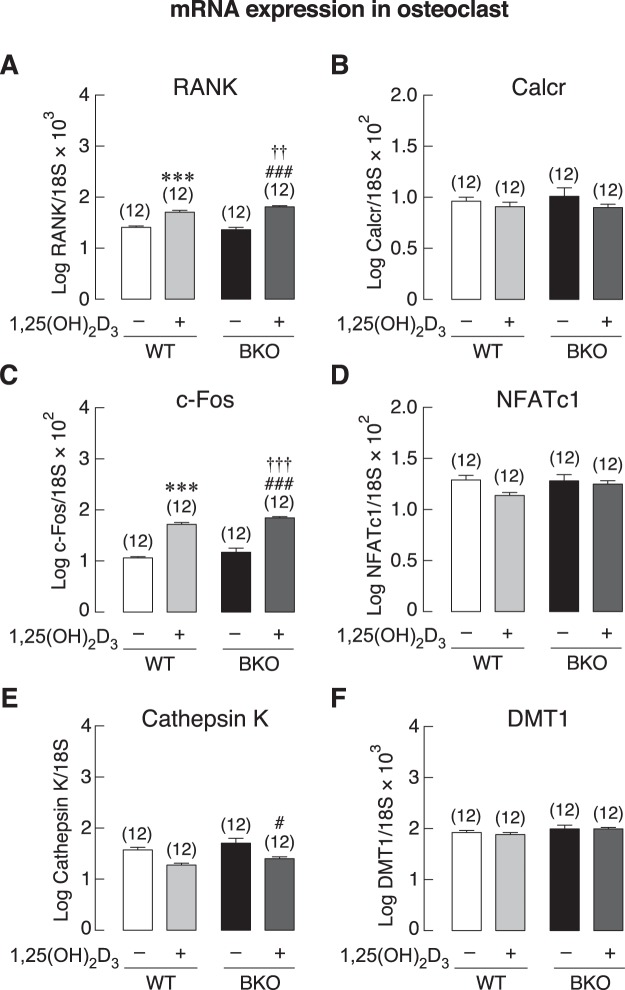
mRNA expression levels of osteoclast function-related genes, i.e., (**A**) RANK, (**B**) calcitonin receptor (Calcr), (**C**) c-Fos, (**D**) NFATc1, and (**E**) cathepsin K, and (**F**) DMT1, in 8 days primary osteoclasts culture of BKO and WT mice. Primary osteoclasts were obtained from 0.5 μg/kg 1,25(OH)_2_D_3_-treated or 3 mL/kg 9:1 propylene glycol-ethanol (vehicle)-treated BKO and WT mice. The 18 S rRNA was a housekeeping gene for normalization. ***P < 0.001 vs. vehicle-treated WT mice (white bar), ^#^*P* < 0.05, ^###^*P* < 0.001 vs. vehicle-treated BKO mice (black bar), ^††^*P* < 0.01, ^†††^*P* < 0.001 vs. 1,25(OH)_2_D_3_-treated WT mice. Numbers in parentheses represent the number of independent samples per group.

The experimental design pertaining to primary osteoclasts is shown in Fig. 5A. Although primary osteoclasts from WT and BKO mice exhibited similar morphology of the multinucleated cells (Fig. 5B), BKO + Veh group had less number of TRAP-positive cells than WT + Veh group (Fig. 5C). When the mice were treated with 1,25(OH)_2_D_3_ as depicted in the timeline (Fig. 5A), the culture from 1,25(OH)_2_D_3_-treated mice showed a significant increase in the numbers of TRAP-positive cells (Fig. 5C). However, the primary osteoclasts from both WT and BKO mice expressed similar mRNA levels of osteoclast function-related genes, i.e., RANK, calcitonin receptor (Calcr), c-Fos, NFATc1, cathepsin K and DMT1 (Fig. 6). After 1,25(OH)_2_D_3_ treatment, only the expression of RANK and c-Fos in BKO-derived osteoclasts were upregulated (Fig. 6).

### High glucose level did not account for the changes in expression of osteoblast- or osteoclast-related markers in BKO-derived primary bone cells

**Figure 7:**
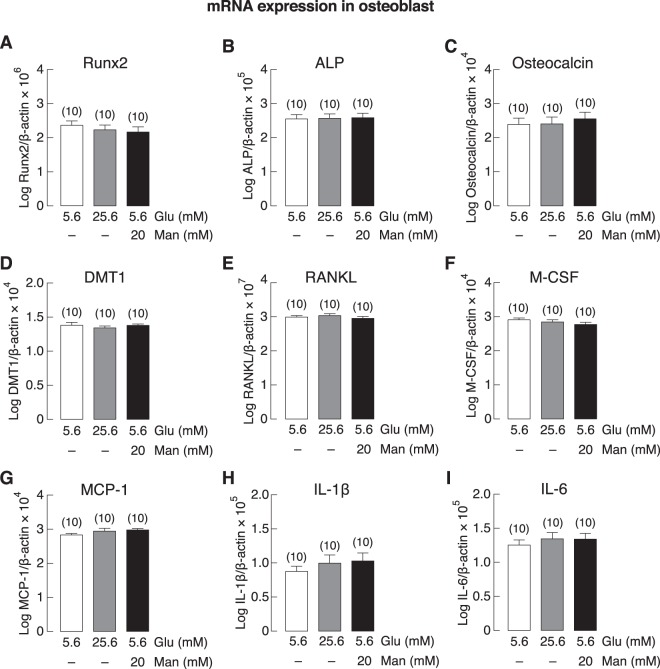
mRNA expression levels of osteoblast differentiation and function-related genes, i.e., (**A**) Runx2, (**B**) ALP, (**C**) osteocalcin, (**D**) DMT1, (**E**) RANKL, (**F**) M-CSF, (**G**) MCP-1, (**H**) IL-1β, and (**I**) IL-6 in primary osteoblasts of WT mice cultured with normal glucose (5.6 mM glucose), high glucose (25.6 mM glucose), or osmotic control (5.6 mM glucose + 20 mM mannitol) for 7 days. β-actin was a housekeeping gene for normalization. Numbers in parentheses represent the number of independent samples per group.

**Figure 8:**
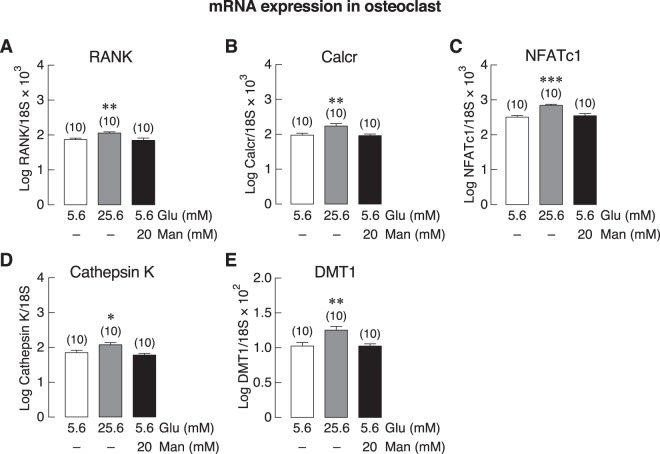
mRNA expression levels of osteoclast function-related genes, i.e., (**A**) RANK, (**B**) calcitonin receptor (Calcr), (**C**) NFATc1, (**D**) cathepsin K, and (**E**) DMT1 in primary osteoclasts of WT mice cultured with normal glucose (5.6 mM glucose), high glucose (25.6 mM glucose), or osmotic control (5.6 mM glucose + 20 mM mannitol) for 7 days. The 18 S rRNA was a housekeeping gene for normalization. *P < 0.05, **P < 0.01, ***P < 0.001 vs. 5.6 mM glucose-treated group (control group; white bar). Numbers in parentheses represent the number of independent samples per group.

Since BKO mice exhibited an elevation of fasting blood glucose (Fig. 1) similar to that observed in several thalassemic patients^2,22,23^, we further explored whether exposure to high extracellular glucose could contribute to osteoblast and osteoclast phenotypes of BKO mice. By using primary bone cells from WT mice, we found that high extracellular glucose of 25.6 mM (with osmolality of 326 mmol/kg water) as well as 5.6 mM glucose + 20 mM mannitol (the same osmolality as 25.6 mM glucose) did not alter the mRNA expression of Runx2, ALP, osteocalcin, DMT1, RANKL, M-CSF, MCP-1, IL-1β or IL-6 in the primary osteoblasts of WT mice (Fig. 7), suggesting that high-glucose condition was not able to turn wild-type osteoblasts to cells with phenotype similar to BKO-derived cells that had lower expressions of Runx2, ALP, and osteocalcin (Fig. 3). On the other hand, 25.6 mM glucose, but not a condition with equivalent osmolality (5.6 mM glucose + 20 mM mannitol), modestly but significantly upregulated the mRNA expression of RANK, Calcr, NFATc1, cathepsin K, and DMT1 in primary osteoclasts (Fig. 8). Thus, high glucose level did not contribute or explain the changes in the expression of osteoblast- or osteoclast-related markers in BKO-derived bone cells.

## Discussion

Calcium and bone metabolism, including intestinal calcium absorption and bone turnover, are affected by a number of factors, such as hormones [e.g., 1,25(OH)_2_D_3_, fibroblast growth factor-23 and prolactin] and pathological conditions (e.g., diabetes mellitus and thalassemia)^3,5,13,24–26^. It was previously believed that thalassemia-associated osteopenia and osteoporosis predominantly resulted from inappropriate release of inflammatory cytokines during ineffective erythropoiesis, iron overload and the resultant chronic inflammation^2^, which could, in turn, suppress the osteoblast function while enhancing the osteoclast activity^3,27^. However, whether the genetic background—i.e., the β-thalassemia genotype—as well as hyperglycemia contributed to bone cell abnormalities remained unclear. Here, we developed a technique to culture osteoblasts from BKO mice and found that the primary osteoblasts from BKO mice manifested lower-than-normal expression of osteoblast-specific genes (e.g., Runx2, ALP and osteocalcin; Fig. 3), which was able to explain the lower bone formation as reported previously^3,28^.

Although ALP expression was downregulated in BKO group as compared with WT group, visual examination confirmed that cells with osteoblastic morphology in BKO group did express ALP, i.e., BKO-derived osteoblasts were also ALP-positive cells despite lower intensity of ALP fluorescent signals (Supplementary Fig. S3). Since primary osteoblast culture is often contaminated with other cell types, such as hematopoietic cells and stromal cells, ALP-positive cell sorting help confirm osteoblast number in primary culture. The present data indicated that both BKO and WT groups had similar number of ALP-positive osteoblasts. On the other hand, BKO osteoclast precursors were less likely to differentiate into mature osteoclasts as compared to WT cells. In other words, BKO genotype was associated with the lower number of TRAP-positive cells in primary osteoclast culture, probably resulted from delayed differentiation from bone marrow cells into TRAP-positive osteoclasts. β-thalassemia was also reported to show delayed bone growth and maturation, e.g., delayed osteoid mineralization^29^, that indicated aberration of bone remodeling.

In primary osteoblast culture, there was lower Runx2 expression in BKO than WT groups (Fig. 3). This could well explain the present bone histomorphometric data (Supplementary Fig. S1) in male BKO mice showing decreases in osteoblast surface, osteoid volume and bone formation rate, which were primary indicators of reduced osteoblast activity and compromised matrix production, respectively, and thus agreeing with the previous data in female thalassemic mice^3,28^. Downregulation of ALP and osteocalcin were also consistent with the lower mineralization activity seen in thalassemic mice^3^. In addition, the BKO osteoblasts showed modest change in the expression of osteoclastogenic factors. Specifically, only MCP-1 expression was upregulated in the primary osteoblasts from BKO mice (Fig. 4), suggesting that the previously observed increases in osteoclast surface and active erosion surface in the tibial metaphysis was not mainly due to the production of osteoblast-derived osteoclastogenic factor. In other words, the enhanced osteoclast activity *in vivo* might have resulted from other circulating osteoclastogenic cytokines, e.g., IL-1α, IL-1β, IL-6, and TNF-α from other sources rather than local cytokine production from neighboring bone cells^3,30^.

Interestingly, 1,25(OH)_2_D_3_ was found to decrease RANKL expression in WT osteoblasts (Fig. 4A). Although it is known that direct exposure to 1,25(OH)_2_D_3_ *in vitro* can enhance osteoblastic RANKL expression, thereby upregulating osteoclastogenesis^31,32^, the *in vivo* effects of 1,25(OH)_2_D_3_ treatment on osteoblasts are much different and somewhat dependent on several factors, such as dose and duration of treatment. Consistent with our study, eldecalcitol (ED-71), a vitamin D derivative, was found to increase serum calcium, bone mineral density (BMD) *in vivo*, and caused a reduction in femoral RANKL mRNA expression, the latter of which, in turn, compromised bone turnover^33^. Alternatively, the downregulated RANKL expression in the present study was probably related to 1,25(OH)_2_D_3_-enhanced osteoblast differentiation. Specifically, RANKL is highly expressed in less differentiated osteoblasts with low ALP expression, and its expression is progressively decreased in ALP-positive mature osteoblasts^34^.

**Figure 9:**
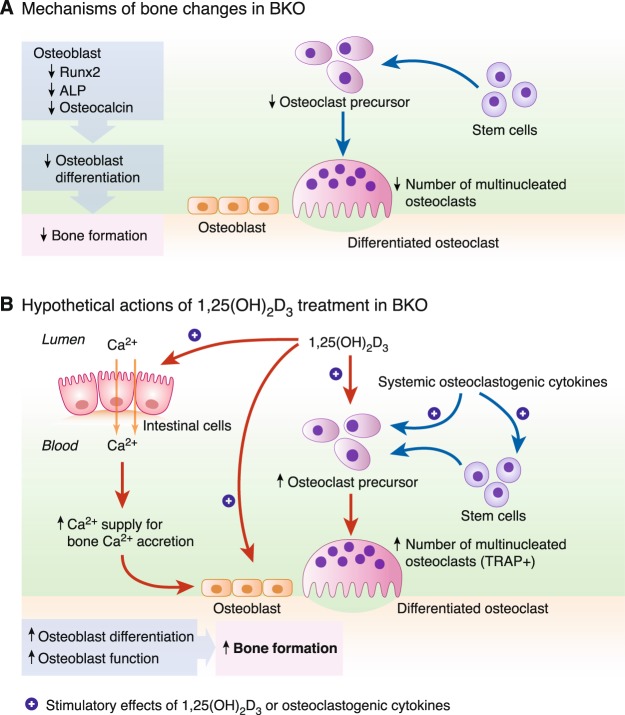
(**A**) A schematic diagram shows possible role of thalassemia-associated osteopenia and osteoporosis by reducing capability of bone cell differentiation *in vitro*. A number of BKO-derived osteoclasts are lower than WT mice. Thalassemia also impairs osteoblast differentiation as shown by decreased expression of Runx2, ALP, and osteocalcin transcripts in BKO-derived primary osteoblasts. (**B**) A schematic diagram shows the hypothetical mechanisms of preceding 1,25(OH)_2_D_3_ treatment in BKO mice that stimulate differentiation and function of osteoblasts and osteoclasts. 1,25(OH)_2_D_3_ directly promotes osteoblast differentiation and function and optimizes osteoclast function to balance bone remodeling, and enhances intestinal calcium absorption, which, in turn, indirectly promotes osteoblast function.

The primary osteoclasts *in vitro* were likely to behave differently from those *in vivo*. We observed herein that the BKO culture had a lower number of TRAP-positive cells than the WT culture, indicating that fewer osteoclast precursors differentiated into active osteoclasts (Fig. 5), which was contrast to the *in vivo* finding (Supplementary Fig. S1). Both BKO and WT osteoclasts also exhibited similar expression levels of osteoclast-specific markers, e.g., RANK, NFATc1 and cathepsin K, all of which were required for osteoclast-mediated bone resorption (Fig. 6). Normally, osteoclasts arise from monocyte precursors to form multinucleated giant cells to resorb bone. The phenotype of macrophage lineage including osteoclasts is determined by the complex mixture of cytokines, metabolites, plasma proteins, and microbial ligands present in the inflammatory milieu^35^. Our previous investigation has reported that thalassemia-induced bone loss is associated with circulating pro-inflammatory cytokines^3^, which markedly enhance osteoclastogenesis and osteoclast activity. Since primary osteoclasts in this study had no longer exposed to serum cytokines, some thalassemia-induced changes might disappear. In addition, osteoclast progenitors (from macrophage/monocyte lineage) *in vivo* are probably exposed to a considerable amount of hematopoietic cell-derived cytokines in bone marrow during ineffective erythropoiesis and bone marrow expansion, leading to more robust enhancement of osteoclast function *in vivo* than *in vitro*. Thus, it was likely that the enhanced differentiation of osteoclasts in BKO mice *in vivo* was activated by certain circulating osteoclastogenic factor(s), e.g., IL-1, and not driven exclusively by their genetic background. Our results further suggested that the thalassemic genotype or genetic background indeed compromised the capabilities of both cell types—particularly in osteoblasts (Fig. 9A)—rather than enhancing the osteoclast function as *in vivo* finding.

1,25(OH)_2_D_3_ was also reported to improve calcium metabolism in thalassemic mice, especially to enhance intestinal calcium absorption to supply more calcium for bone formation^5^. Basically, 1,25(OH)_2_D_3_ directly affects bone cells by activating osteoblast activities and induces bone formation or mineralization^36^. However, paradoxical actions of 1,25(OH)_2_D_3_ on bone are found in both *in vivo* and *in vitro* studies^37^. In the present study, we evaluated differentiation markers of osteoblasts and osteoclasts derived from 1,25(OH)_2_D_3_-treated mice. The 1,25(OH)_2_D_3_ was injected into BKO mice for 7 days before harvesting the cells. The expression levels of osteoblast and osteoclast differentiation markers in osteoblasts were mostly upregulated by the *in vivo* 1,25(OH)_2_D_3_ pretreatment (Figs 3–4). Interestingly, similar findings were previously observed in a primary culture even when the cells were no longer exposed to the BKO circulating factors in the plasma, which suggested that cell response in this situation was due to the well-known epigenetic effects of 1,25(OH)_2_D_3_^38,39^. Specifically, 1,25(OH)_2_D_3_ was able to modulate gene promoter methylation and histone acetylation, as shown earlier in breast cancer cells and certain hematopoietic cells^38,40^. Both types of epigenetic regulation often persisted after cell division and probably continued into the descendent cells *in vitro*^41–43^.

Although 1,25(OH)_2_D_3_ was herein found to stimulate osteoclastogenesis *ex vivo*, it is difficult to extrapolate its systemic responses *in vivo*. Some *in vivo* studies showed the opposite action of 1,25(OH)_2_D_3_ with decreased osteoclast numbers and TRAP-positive osteoclasts^44,45^. The final outcome of 1,25(OH)_2_D_3_ action probably depends on the dosage and circulating humoral factors^37^. In our BKO model, we hypothesized that 1,25(OH)_2_D_3_ could help alleviate bone loss by activating intestinal calcium absorption to increase calcium supply and optimizing osteoblast and osteoclast activities to mediate balance of bone remodeling and to maintain proper bone metabolism^46^ (Fig. 9B). However, further investigation was required to reveal the underlying mechanism of the responsiveness of osteoblast- and osteoclast-related genes under the regulation of 1,25(OH)_2_D_3_ in thalassemia.

Besides the known circulating cytokines, high plasma glucose as well as DM might also contribute to bone cell malfunction and bone loss in thalassemic patients. Here, BKO mice also exhibited high plasma glucose, impaired insulin tolerance with abnormal HOMA index (Fig. 1). Several DM-related conditions—i.e., excessive production of advanced glycation end products (AGEs) and chronic inflammation—have been reported to suppress osteoblast-mediated bone formation and accelerate osteoclast-mediated bone resorption (for review, please see^13,47^). However, it is not possible to mimic the exact BKO conditions (e.g., time-dependent changes in glucose levels with inappropriately low insulin level) in cell culture system. Therefore, we designed the experiment to demonstrate whether high extracellular glucose could convert WT bone cells to BKO-like cells. It was found that primary osteoblasts that had been exposed to high glucose did not show any change comparable to those observed in BKO-derived osteoblasts (Fig. 7). On the other hand, high glucose significantly upregulated osteoclast differentiation markers (i.e., RANK, NFATc1, cathepsin K, calcitonin receptor), and DMT1 (Fig. 8), consistent in part with the increased bone resorption that previously reported *in vivo*^28^. It is still unclear whether hyperglycemia contributes to BKO characteristics or structural bone change *in vivo*. A previous *in vivo* study demonstrated that hyperglycemia alone decreased osteoblast and osteoclast activities^11^, suggesting that high glucose can compromise bone turnover *in vivo*. Nevertheless, the present finding revealed that high glucose had no significant effects on the changes in bone marker expression in WT osteoblasts *in vitro*, while it upregulated osteoclast-specific gene expression.

We also found that mRNA expression of insulin receptor decreased after exposure to 1,25(OH)_2_D_3_. Upregulation and downregulation of insulin receptor expression is considered as a sign of increased and decreased insulin resistance, respectively^48,49^ (i.e., a decrease in insulin resistance requires less receptor number). Since vitamin D is able to improve insulin sensitivity (i.e., decreased insulin resistance) in various cell types, such as skeletal muscle cells and bone marrow cells^50,51^, we hypothesized that vitamin D should downregulate insulin receptor expression in osteoblasts, as confirmed by the data in Fig. 3B.

Adequate calcium supply is essential for bone formation, particularly the mineralizing process. Although the impaired intestinal calcium absorption contributed to deterioration of bone metabolism in BKO mice, serum calcium of BKO mice might be maintained within a normal range by actions of several hormones, such as parathyroid hormone (PTH)^52,53^. 1,25(OH)_2_D_3_ treatment probably increases serum calcium but it should not exceed the normal range due to rapid distribution of serum calcium to many tissues, including bone. Harada *et al*. (2012)^33^ have reported that exogenous vitamin D derivative supplement slightly increased serum calcium; however, the level of total calcium remained within the normal range of 2.25–2.5 mM, which is crucial for the functions of excitable cells, such as cardiac myocyte and neuron. The underlying mechanisms of thalassemia-induced impairment of calcium transport was unclear, but it was postulated to result from a decrease in 1,25(OH)_2_D_3_ responsiveness in the intestine as well as low circulating calciotropic hormone levels (Fig. 9B). Although we have previously reported that the 7-day 1,25(OH)_2_D_3_ injection could significantly enhance the duodenal calcium absorption in BKO mice^5^, whether restoration of calcium absorption is able to normalize bone turnover remains to be investigated.

In conclusions, the present BKO mice exhibited several signs of impaired bone and calcium metabolism, such as lower rate of the duodenal calcium transport and aberrant bone cell functions. We have provided evidence that BKO genotype was associated with abnormalities of both osteoblasts and osteoclasts, which could be partially restored by *in vivo* 1,25(OH)_2_D_3_ pretreatment. However, prolonged exposure to high extracellular glucose *in vitro* was likely to enhance the expression of osteoclast differentiation-related genes but not that of osteoblast differentiation-related genes, and could not turn WT cells to BKO-like cells. Although more functional studies, such as *in vitro* mineralization study, are required to confirm the conclusions, our results are able to support the beneficial effects of 1,25(OH)_2_D_3_ on thalassemia-associated osteopenia and osteoporosis.

## Materials and Methods

### Animals and experimental design

Seven-week-old male and female C57BL/6 wild-type (WT) and hemizygous β-globin knockout thalassemic (BKO) mice with body weight of 15–30 g were obtained from the Thalassemia Research Center, Institute of Molecular Biosciences, Mahidol University. The experimental protocol was approved by the Institutional Animal Care and Use Committee (IACUC), Faculty of Science, Mahidol University (protocol no. MUSC56–012–274), and all experiments were performed in accordance with relevant guidelines and regulations.

After 7-day acclimatization, fasting blood glucose was measured at 8 and 15 weeks of age to indicate glucose homeostasis. Insulin tolerance test (ITT) was performed in 8-week-old mice to evaluate whole body responsiveness to insulin. In ITT experiment, after fasting for 5 h, mice were intraperitoneally injected with 0.45 U/kg insulin (Humulin R, Eli Lilly and Company, IN, USA). Blood sample was collected from tail vein at 0, 15, 30, 60, and 120 min for measuring blood glucose levels by Accu-Chek Active Test Strips. Fasting blood glucose and insulin of 15-week-old mice were used to determine homeostatic model assessment (HOMA), an index of insulin resistance. Fasting insulin level was measured by ultra-sensitive mouse insulin ELISA kit (catalog no. 90080; Crystal Chem, Downers Grove, IL, USA). Furthermore, to determine the intestinal calcium transport of BKO mice, the duodenal tissue was studied *ex vivo* by Ussing chamber technique.

To investigate the potential effect of 1,25(OH)_2_D_3_ treatment in restoring bone cell function, 8-week-old BKO mice and their age-matched WT mice were subcutaneously injected with vehicle (Veh; 3 mL/kg 9:1 propylene glycol-ethanol) or 0.5 μg/kg 1,25(OH)_2_D_3_ (catalog no. 71820; Cayman Chemical, Ann Arbor, MI, USA) for 7 days. After euthanasia, tibiae and femora were collected for isolating primary osteoblasts or osteoclast precursors. Osteoblasts and osteoclasts were cultured for 8 days for mRNA expression study (Figs 3A and 5A). In some experiments, 8-week-old male WT and BKO mice were subcutaneously injected with two doses of calcein (10 mg/kg body weight) on day 7 and 1 prior to euthanasia to label bone for dynamic histomorphometric study. The tibiae were then collected for bone histomorphometry.

In order to investigate the contribution of high glucose on bone defect of BKO mice, primary osteoblasts and osteoclasts were obtained from 9-week-old WT mice. After plating for 1 day, cells were cultured in α-MEM containing normal glucose (5.6 mM glucose; osmolality 305 mmol/kg water), high glucose (25.6 mM glucose; osmolality 326 mmol/kg water), or osmotic control (5.6 mM glucose + 20 mM mannitol; osmolality 325 mmol/kg water) for 7 days. Cells were harvested for total RNA preparation and mRNA expression study.

### Bone histomorphometry

Bone histomorphometric analysis was performed as previously described^3^ using a computer-assisted OsteoMeasure System version 4.1 (OsteoMetrics, Atlanta, GA) to re-confirm the presence of compromised osteoblast activity. After clearing adherent tissue, the tibiae were dehydrated in 70, 95, and 100% vol/vol ethanol for 3, 3, and 3 days, respectively. Bone specimens were later embedded in methyl methacrylate resin at 42 °C for 48 h. The resin-embedded tibiae were longitudinally cut into 5- or 10-μm-thick sections with a microtome equipped with a tungsten carbide blade (model RM2255; Leica, Nussloch, Germany). All parameters were evaluated at secondary spongiosa, which was the trabecular part of tibial metaphysis. Static bone parameters [i.e., osteoblast surface normalized by bone surface (Ob.S/BS, %), osteoid surface normalized by BS (OS/BS, %), osteoid volume normalized by tissue volume (OV/TV, %), osteoclast surface normalized by BS (Oc.S/BS, %) and active erosion surface normalized by BS (aES/BS, %)] were examined under a light microscope (model BX51TRF; Olympus, Tokyo, Japan) from 5-μm-thick sections stained with Goldner’s trichrome. Dynamic bone parameters [i.e., mineral apposition rate (MAR, μm/day), double labeled surface (dLS/BS, %) and bone formation rate (BFR/BS, μm^3^/μm^2^/day)] were analyzed from double lines of calcein in 10-μm-thick unstained sections under a fluorescent microscope (model BX51TRF; Olympus).

### Primary osteoblast culture

Primary osteoblasts were prepared from long bones (tibiae and femora) of 9-week-old mice, which were treated with either vehicle (Veh) or 1,25(OH)_2_D_3_ for 7 days before bone cell isolation. Primary osteoblast culture protocol was modified from the method of Wongdee *et al*.^54^. Cells were maintained in α-MEM with 30% fetal bovine serum (FBS) for 8 days. In high glucose experiment, 20 mM glucose was added into α-MEM to mimic high glucose concentration while 20 mM mannitol was used as osmotic control for high glucose condition. Cells were cultured in normal glucose, high glucose, or osmotic control for 7 days after plating. Immunolocalization and flow cytometry for ALP-positive cells were used to determine successful of primary osteoblast isolation.

### Primary osteoclast culture

Primary osteoclasts were derived from bone marrow cells of tibiae and femora. Protocol for primary osteoclast isolation was modified from the method of Marino *et al*.^55^. In high glucose experiment, cells were cultured in α-MEM containing normal glucose, high glucose, or mannitol control for 7 days after plating. The multinucleated osteoclasts were clearly observed at days 7–8. Tartrate-resistant acid phosphatase (TRAP) staining and TRAP-positive cell count were used to determine the success of osteoclast isolation.

### Immunofluorescence

The confluent osteoblasts were plated at 3 × 10^5^ cells/well on glass coverslips in 12-well plates (Corning, NY, USA). Cells were fixed by 4% paraformaldehyde and nonspecific binding was blocked with 5% normal donkey serum (catalog no. 017-000-121; Jackson Immuno Research, PA, USA) in phosphate-buffered saline (PBS). Cells were then incubated with 10 μg/mL goat polyclonal primary antibody against ALP (catalog no. AF2910; R&D Systems, MN, USA) at 4 °C overnight. For negative control, cells were incubated with 10 μg/mL polyclonal goat IgG control (catalog no. AB-108-C; R&D Systems). ALP protein expression was detected by incubating with 1:100 donkey anti-goat IgG conjugated with fluorescein (FITC) (catalog no. 705-095-147; Jackson Immuno Research) for 1 h in dark condition and mounted with anti-fade mounting medium with DAPI (catalog no. H-1200; Vector, Burlin-game, CA, USA). The coverslips were visualized under a confocal microscope (model FV10i-DOC; Olympus, Tokyo, Japan). These ALP antibody and normal goat IgG (isotype control) were further used for flow cytometry.

### Flow cytometry

The confluent primary osteoblasts were collected and incubated with 5% normal donkey serum for blockade of nonspecific binding. Cells were incubated with either 2.5 μg primary antibody against ALP or normal goat IgG control (isotype control), followed by FITC-conjugated secondary antibody (1:100) for 1 h at 4 °C. FITC-labeled cells were analyzed at 515–545 nm by flow cytometer (model BD FACSCanto; BD Bioscience, NJ, USA). Forward scatter (FSC) and side scatter (SSC) gating were set appropriately for the cells. Rat osteoblast-like UMR-106 cell line [catalog no. CRL-1661; American Type Culture Collection (ATCC), VA, USA] was used as positive control for ALP-positive cell analysis.

### Tartrate-resistant acid phosphatase (TRAP) staining and quantification of TRAP-positive cells

Bone marrow cells were plated at 1 × 10^5^ cells/well in 96-well plates and cultured in α-MEM containing osteoclastogenic factors for 8 days. Cells were fixed with 10% neutral buffer formalin and incubated with TRAP chromogenic substrate (catalog no. PMC-AK04F-COS; Cosmo Bio, Tokyo, Japan) at 37 °C for 60 min. TRAP-positive multinucleated osteoclasts containing ≥3 nuclei were visualized and manually counted under an inverted microscope (model IX83ZDC; Olympus) as previously described^56,57^. The number of cells was represented as TRAP-positive cells per well.

### Total RNA extraction and quantitative real-time PCR (qRT-PCR)

Primary osteoblasts and bone marrow cells were plated at 3 × 10^5^ and 5 × 10^5^ cells/well, respectively, in 6-well plates. Total RNA extraction and cDNA synthesis were performed as previously described^58^. The primers used in this study are shown in Supplementary Table S1. The qRT-PCR and melting curve analyses were performed with SsoFast EvaGreen Supermix (catalog no. 172–5204; Bio-rad, CA, USA) and QuantStudio 3 Real-Time PCR system (Applied Biosystems, MA, USA). Mouse β-actin or 18 S rRNA were used as housekeeping genes for primary osteoblasts and osteoclasts, respectively. Changes in gene expression were calculated from the threshold cycles (C_t_).

### Transepithelial calcium transport

Determination of *ex vivo* intestinal calcium transport was performed as previously described^59^. Briefly, the duodenum was cut longitudinally to expose the mucosa and was rinsed by isotonic bathing solution containing (in mmol/L) 118 NaCl, 4.7 KCl, 1.1 MgCl_2_, 1.25 CaCl_2_, 23 NaHCO_3_, 12 d-glucose, and 2 mannitol (all purchased from Sigma). The intestinal tissue was then mounted in an Ussing chamber and bathed on both sides of the hemichambers with an isotonic bathing solution for 10 min. In the mucosal hemichamber, the solution was changed to the bathing solution containing a radioactive tracer, ^45^Ca (initial amount of 0.451 Ci/mL, final specific activity of 90 mCi/mol; catalog no. NEZ013; PerkinElmer, Boston, MA, USA). Unidirectional calcium flux (*J*_H→C_, nmol/h/cm^2^) from the hot side (H; mucosal side) to the cold side (C; serosal side) was calculated by Eqs 1 and 2:

1 $${J}_{{\rm{H}}\to {\rm{C}}}={R}_{{\rm{H}}\to {\rm{C}}}/({S}_{{\rm{H}}}\times A)$$

2 $${S}_{{\rm{H}}}={C}_{{\rm{H}}}/{C}_{{\rm{T}}{\rm{o}}}$$

where *R*_H→C_ is the rate of ^45^Ca appearance in the cold side (cpm/h); *S*_H_ is the specific activity of the hot side (cpm/ nmol); *A* is the surface area of the tissue (cm^2^); *C*_H_ is the mean radioactivity of the hot side (cpm); and *C*_To_ is the total calcium content in the hot side (nmol). ^45^Ca radioactivity was analyzed by a liquid scintillation spectrophotometer (model Tri-Carb 3100; Packard, Meriden, CT, USA). Due to the same calcium concentration of 1.25 mmol/L in both hemichambers (i.e., the absence of transepithelial calcium gradient), the measured calcium flux represented the active calcium transport in the mucosal-to-serosal direction.

### Statistical analysis

Unless otherwise specified, the results are presented as means ± standard error (SE). Two sets of data were compared using unpaired Student’s *t*-test. One-way analysis of variance (ANOVA) with Newman–Keuls multiple comparisons test was used for multiple sets of independent data. The level of significance for statistical tests was *P* < 0.05. All data were analyzed by GraphPad Prism 5 (GraphPad Software, San Diego, CA, USA).

## Supplementary information

Supplementary information
