# Supplementary material for: Responses of primary osteoblasts and osteoclasts from hemizygous β-globin knockout thalassemic mice with elevated plasma glucose to 1,25-dihydroxyvitamin D3
Source: Sci Rep. 2019 Sep 27;9:13963. doi: 10.1038/s41598-019-50414-7 (PMC6765013; doi:10.1038/s41598-019-50414-7)
Supplement: Supplementary file 1 — Supplementary information [file 41598_2019_50414_MOESM1_ESM.pdf]

Supplementary Information

Responses of primary osteoblasts and osteoclasts from hemizygous β-globin knockout thalassemic mice with elevated plasma glucose to 1,25-dihydroxyvitamin D_3

Narattaphol Charoenphandhu[1,2,3,4], Ratchaneevan Aeimlapa[1,2], Supagarn Sooksawanwit[1,2], Jirawan Thongbunchoo[2], Jarinthorn Teerapornpuntakit[2,5], Saovaros Svasti[6], Kannikar Wongdee[2,7,]*

[1] Department of Physiology, Faculty of Science, Mahidol University, Bangkok, Thailand
[2] Center of Calcium and Bone Research (COCAB), Faculty of Science, Mahidol University, Bangkok, Thailand
[3] Institute of Molecular Biosciences, Mahidol University, Nakhon Pathom, Thailand
[4] The Academy of Science, The Royal Society of Thailand, Dusit, Bangkok, Thailand
[5] Department of Physiology, Faculty of Medical Science, Naresuan University, Phitsanulok, Thailand
[6] Thalassemia Research Center, Institute of Molecular Biosciences, Mahidol University, Nakhon Pathom, Thailand
[7] Faculty of Allied Health Sciences, Burapha University, Chonburi, Thailand

***Correspondence to:** kannikar@go.buu.ac.th

Table S1. *Mus musculus* primers used in real-time PCR

Gene	Accession no.	Primer (Forward/Reverse)	Product size (bp)	Annealing temperature (°C)	Bone related-function
Osteoblast-derived osteoclastogenic factors					
RANKL	NM_011613	5'-CGCTCTGTTCCTGTACTTTCG-3'<br>5'-CTCTCCAGAGTCGAGTCCTGC-3'	126	57	• Inducing osteoclastogenesis
M-CSF	NM_007778	5'-GACCCTCGAGTCAACAGAGC-3'<br>5'-TGTCAGTCTCTGCCTGGATG-3'	236	58	
MCP-1	NM_011333	5'-AGGTCCCTGTCATGCTTCTG-3'<br>5'-TCTGGACCCATTCCTTCTTG-3'	249	55	• Recruitment of osteoclast precursors
IL-1β	NM_008361	5'-GGGCCTCAAAGGAAAGAATC-3'<br>5'-TACCAGTTGGGGAACTCTGC-3'	183	54	• Promoting osteoclastogenesis and osteoclast activity
IL-6	NM_031168	5'-AGTTGCCTTCTTGGGACTGA-3'<br>5'-TCCACGATTTCCCAGAGAAC-3'	159	56	
Osteoblast differentiation markers					
ALP	NM_001287172	5'-TCTCCAGACCCTGCAACCTC-3'<br>5'-CATCCTGAGCAGACCTGGTC-3'	150	58	• Marker of early osteoblast differentiation
Osteocalcin	NM_001305448	5'-ACGGTATCACTATTTAGGACCTGTG-3'<br>5'-ACTTTATTTTGGAGCTGCTGTGAC-3'	141	57	• Marker of late osteoblast differentiation
Runx2	NM_001146038	5'-AGGGACTATGGCGTCAAACA-3'<br>5'-GGCTCACGTCGCTCATCTT-3'	137	57	• Transcription factor inducing osteoblast differentiation
Osteoclast-related markers					
RANK	NM_009399	5'-CATCTTCGGCGTTTACTACAGG-3'<br>5'-TCCACTTAGACTACTGCAAGCA-3'	91	57	• Receptor for RANKL
Cathepsin K	NM_007802	5'-GAACGAGAAAGCCCTGAAGAGA-3'<br>5'-TATCGAGTGCTTGCTTCCCTTC-3'	190	57	• Protease enzyme involved in bone resorption
Calcr	NM_001355192	5'-CGGACTTTGACACAGCAGAA-3'<br>5'-GTCACCCTCTGGCAGCTAAG-3'	247	55	• Marker of osteoclast differentiation
NFATc1	NM_001164112	5'-CCGTTGCTTCCAGAAAATAACA-3'<br>5'-TGTGGGATGTGAACTCGGAA-3'	152	54	• Stimulating osteoclast differentiation
c-Fos	NM_010234	5'-CCAGTCAAGAGCATCAGCAA-3'<br>5'-AAGTAGTGCAGCCCGGAGTA-3'	247	55	• Transcription factor activating osteoclast differentiation
Iron transporter					
DMT1	NM_001146161	5'-TCAGAGCTCCACCATGACTG-3'<br>5'-TGTGAACGTGAGGATGGGTA-3'	235	57	• Initiating iron uptake
Insulin receptor					
INSR	NM_010568	5'-TCCTGGATTCTGTGGAGGAC-3'<br>5'-ATGGTTGGGCAAACTTTCTG-3'	178	54	
Housekeeping gene					
β-actin	NM_007393	5'-CCAGGTCATCACTATTGGCA-3′<br>5'-ACCACCAGACAGCACTGTGTT-3′	172	54	

RANKL, receptor activator of nuclear factor-κB ligand; M-CSF, macrophage colony-stimulating factor; MCP-1, monocyte chemoattractant protein-1; IL-1β, interleukin-1beta; IL-6, interleukin-6; ALP, alkaline phosphatase; Runx2, *runt*-related transcription factor-2; RANK, receptor for receptor activator of nuclear factor-κB ligand; Calcr, calcitonin receptor; NFATc1, nuclear factor of activated T-cells c1; DMT1, divalent metal ion transporter-1; INSR, insulin receptor

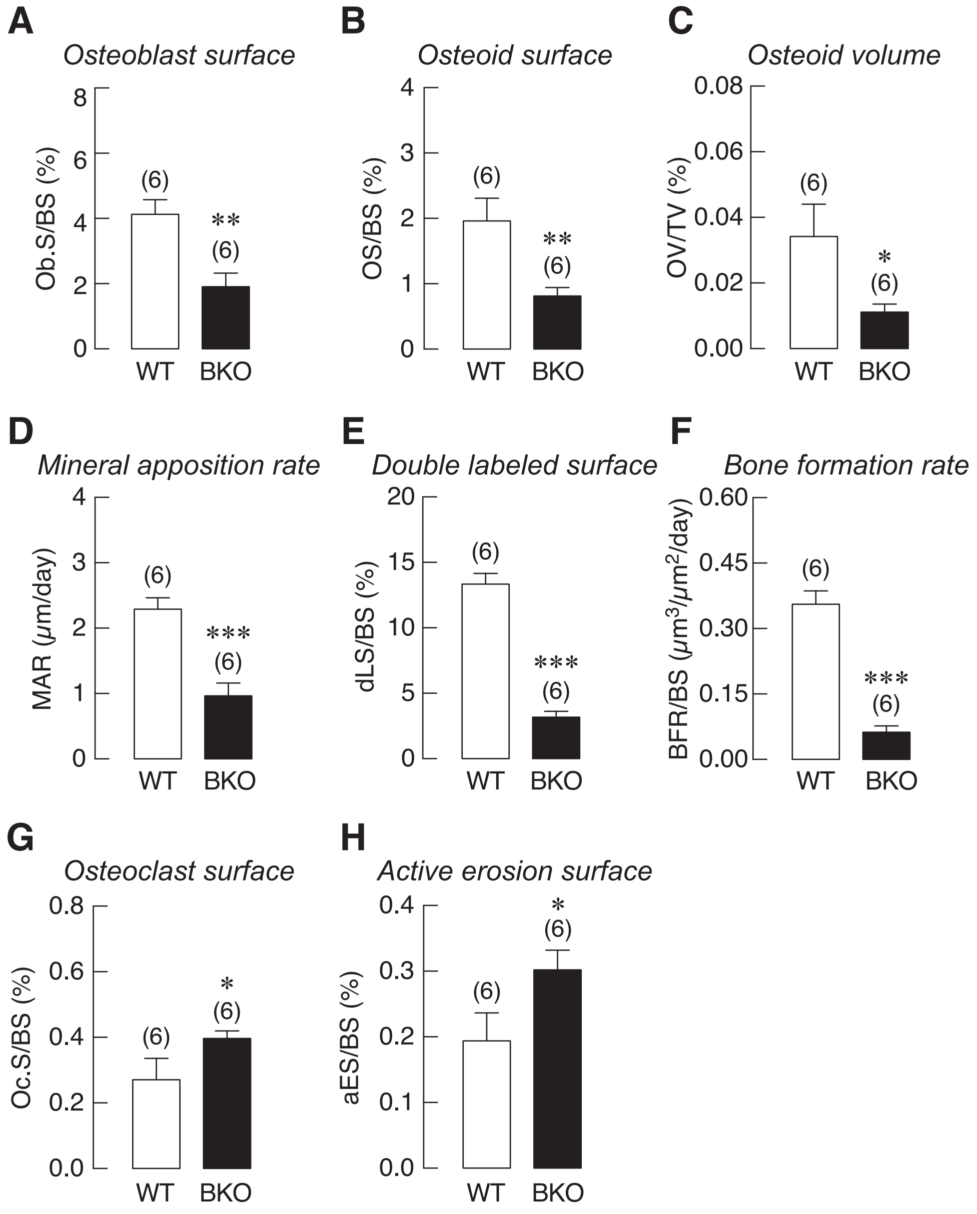

Figure S1: Bone formation- and resorption-related parameters in the proximal tibial metaphyses of 2-month-old male BKO mice. *A*: osteoblast surface (Ob.S) normalized by bone surface (BS), *B*: osteoid surface (OS) normalized by BS, *C*: osteoid volume (OV) normalized by tissue volume (TV), *D*: mineral apposition rate (MAR), *E*: double labeled surface (dLS) normalized by BS, *F*: bone formation rate (BFR) normalized by BS, *G*: osteoclast surface (Oc.S) normalized by BS, *H*: active erosion surface (aES) normalized by BS. Numbers of animals are shown in the parentheses. *$P < 0.05$, **$P < 0.01$, ***$P < 0.001$ vs. age-matched WT group.

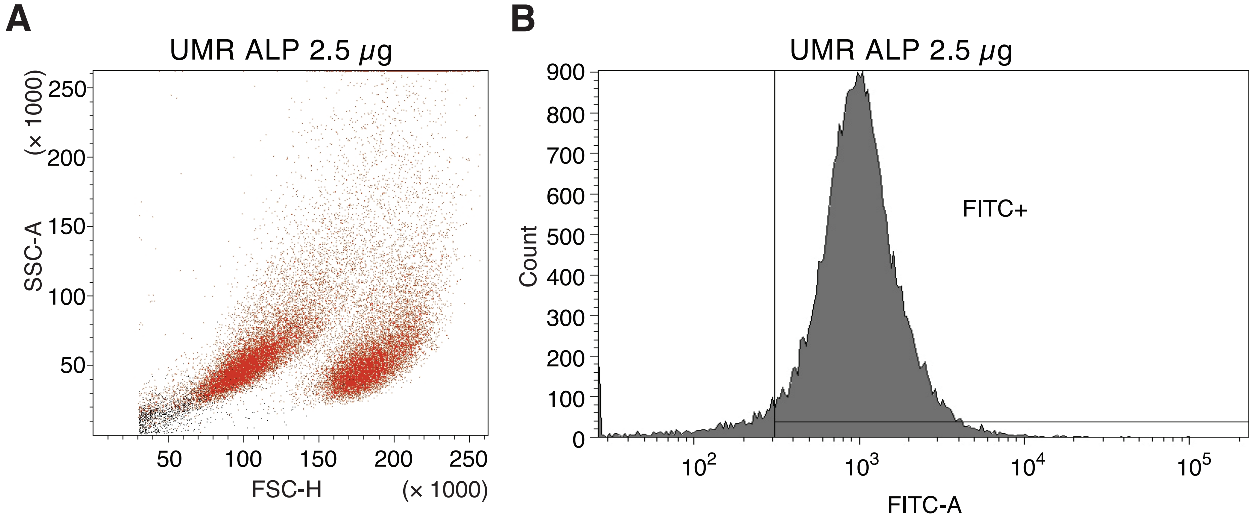

Figure S2: (*A*) Forward scatter (FSC) and side scatter (SSC), and (*B*) histogram of fluorescein (FITC)-conjugated alkaline phosphatase (ALP) in UMR-106 cells as analyzed by flow cytometry.

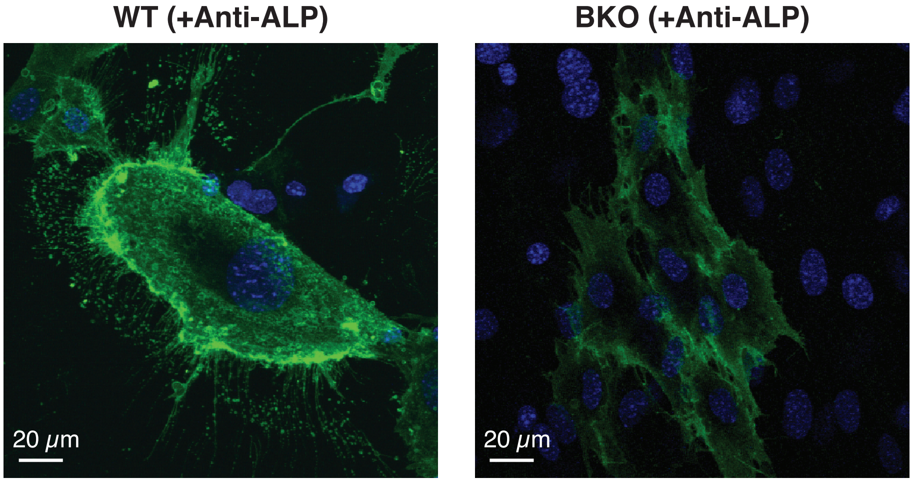

Figure S3: Representative immunofluorescent photomicrographs show the expression and localization of ALP protein in primary osteoblasts isolated from wild-type (WT) and BKO mice (scale bars, 20 μm). The primary osteoblasts were plated on a coverslip and incubated with primary antibody against ALP (+Anti-ALP). The cells were stained for ALP (green) and nuclei (blue).
